# Supplementary material for: Imaging low-energy positron beams in real-time with unprecedented resolution
Source: Sci Rep. 2023 Oct 28;13:18526. doi: 10.1038/s41598-023-45588-0 (PMC10613302; doi:10.1038/s41598-023-45588-0)
Supplement: Supplementary file 1 — Supplementary Information. [file 41598_2023_45588_MOESM1_ESM.zip › SupplementaryMaterials/SupplementaryMaterials_Index.pdf]

Imaging low-energy positron beams in real-time with unprecedented resolution – Supplementary materials index.

`AdditionalLayerRemoval.png`	Phosphor-less detection of positrons with a sensor in which the bottom left corner has been further scraped to remove the wire grid.
`DirectDetections1.png`<br>`DirectDetections2.png`<br>`DirectDetections3.png`<br>`DirectDetections4.png`<br>`DirectDetections5.png`	10 keV beam imaged with a modified sensor, without the phosphor coating. On the sensor surface a 4 µm thick tungsten wire.
`DirectDetection_15keV.png`	15 keV beam imaged with a modified sensor, without the phosphor coating. On the sensor surface a 4 µm thick tungsten wire.
`DirectDetection_AlFoil_SingleFrame.png`	Imaging of a 10 keV beam with a sensor in which only the central area has been scraped to remove Bayer filter and mirolens array. A wedge of aluminun foil has been superimposed in the bottom left corner, partially shadowing the sensor.
`DirectDetection_AlFoil_300FrameSum.png`	Imaging of a 10 keV beam with a sensor in which only the central area has been scraped to remove Bayer filter and mirolens array. A wedge of aluminun foil has been superimposed in the bottom left corner, partially shadowing the sensor. The image has been produced by adding together 300 separate acquisitions
`PartiallyDebayeredSensor1.png`<br>`PartiallyDebayeredSensor2.png`	Optical microscope imaging of the ege between a portion of the sensor where the Bayer filter and microlens array has been removed (yellow-orange-pink) and a pristine area (olive-navy-bordeaux).
`PhosphorDetection1.png`<br>`PhosphorDetection2.png`<br>`PhosphorDetection3.png`<br>`PhosphorDetection4.png`<br>`PhosphorDetection5.png`	10 keV beam imaged with a modified sensor coated with phosphors. The sensor is partially shaded by a tungsten wire.
`RegisterConfiguration.csv`	Configuration of the sensor registers used during the experiments reported in the article.
