# Supplementary figures and images for: Imaging low-energy positron beams in real-time with unprecedented resolution
Source: Sci Rep. 2023 Oct 28;13:18526. doi: 10.1038/s41598-023-45588-0 (PMC10613302; doi:10.1038/s41598-023-45588-0)

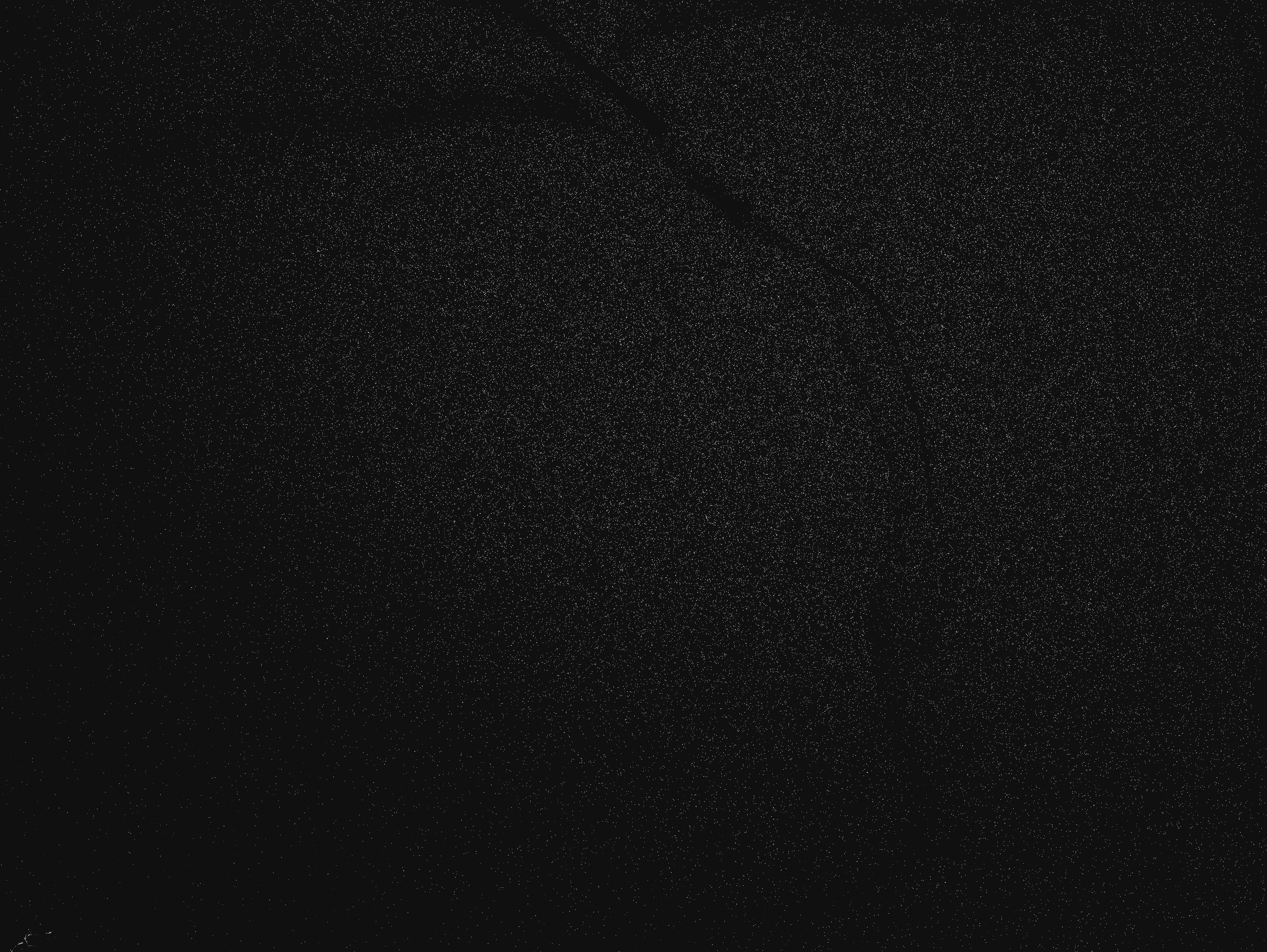

Supplement: Supplementary file 1 — Supplementary Information. [file 41598_2023_45588_MOESM1_ESM.zip › SupplementaryMaterials/DirectDetection1.png]

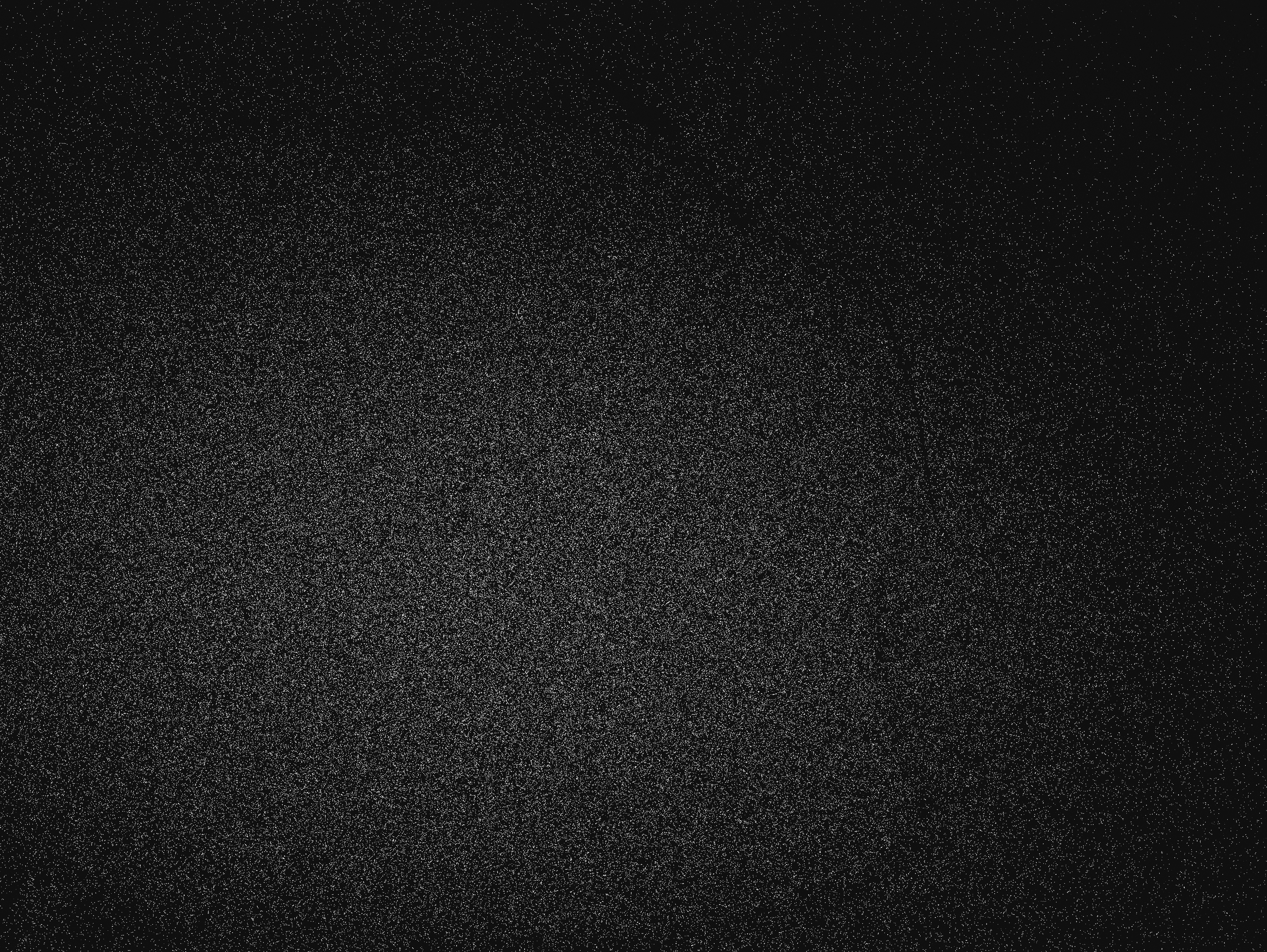

Supplement: Supplementary file 1 — Supplementary Information. [file 41598_2023_45588_MOESM1_ESM.zip › SupplementaryMaterials/DirectDetection_15keV.png]

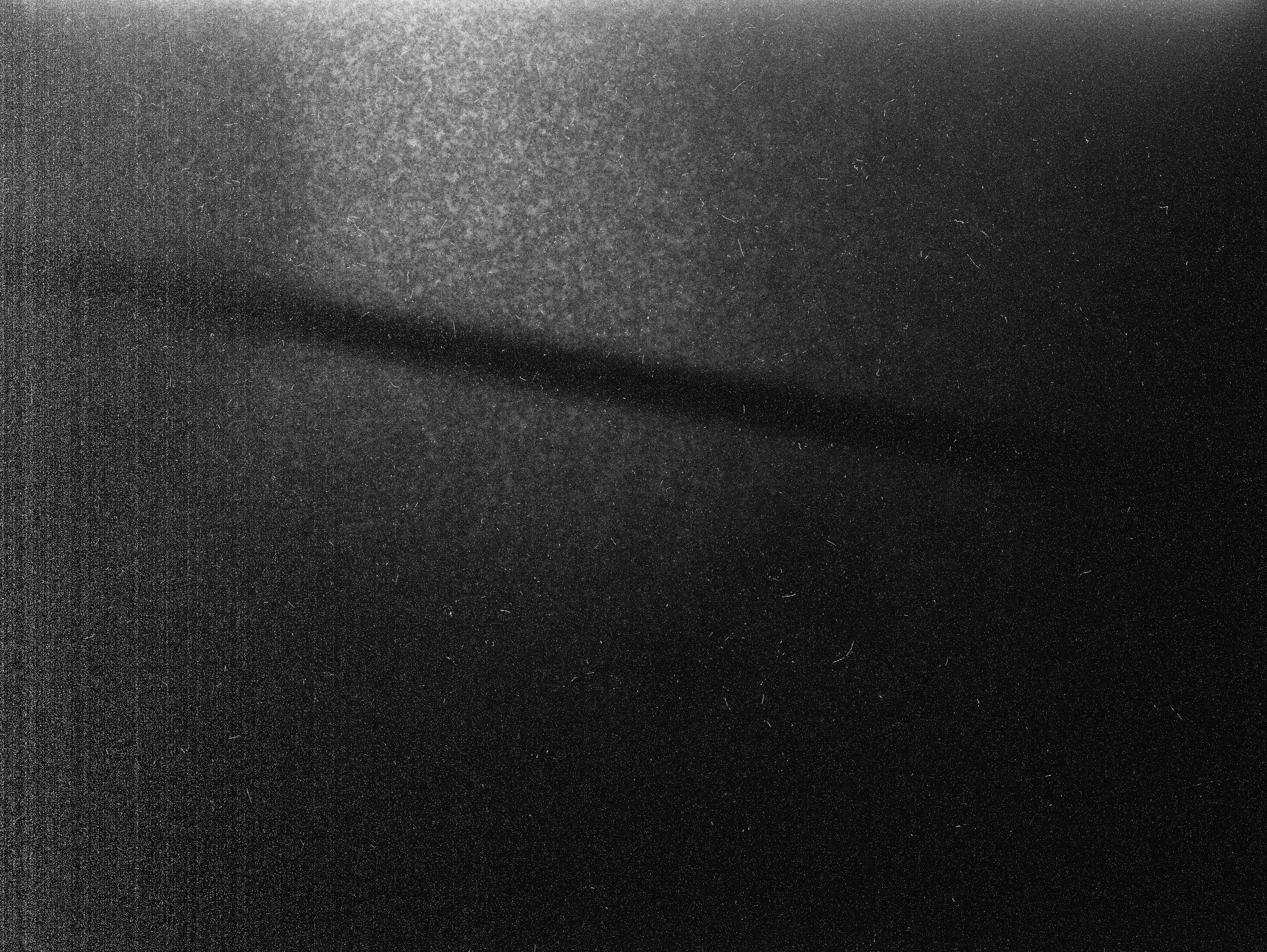

Supplement: Supplementary file 1 — Supplementary Information. [file 41598_2023_45588_MOESM1_ESM.zip › SupplementaryMaterials/PhosphorDetection1.png]

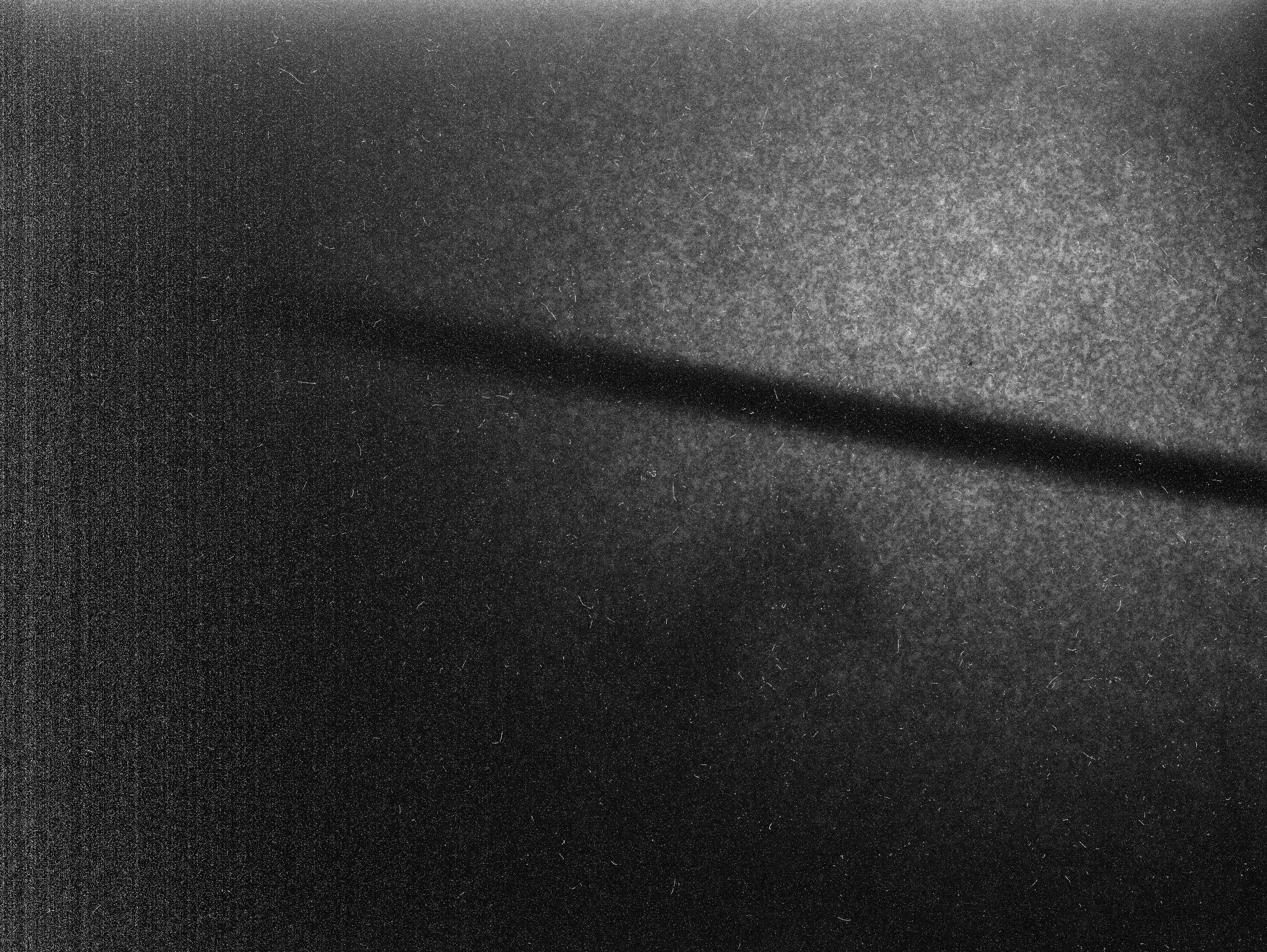

Supplement: Supplementary file 1 — Supplementary Information. [file 41598_2023_45588_MOESM1_ESM.zip › SupplementaryMaterials/PhosphorDetection2.png]

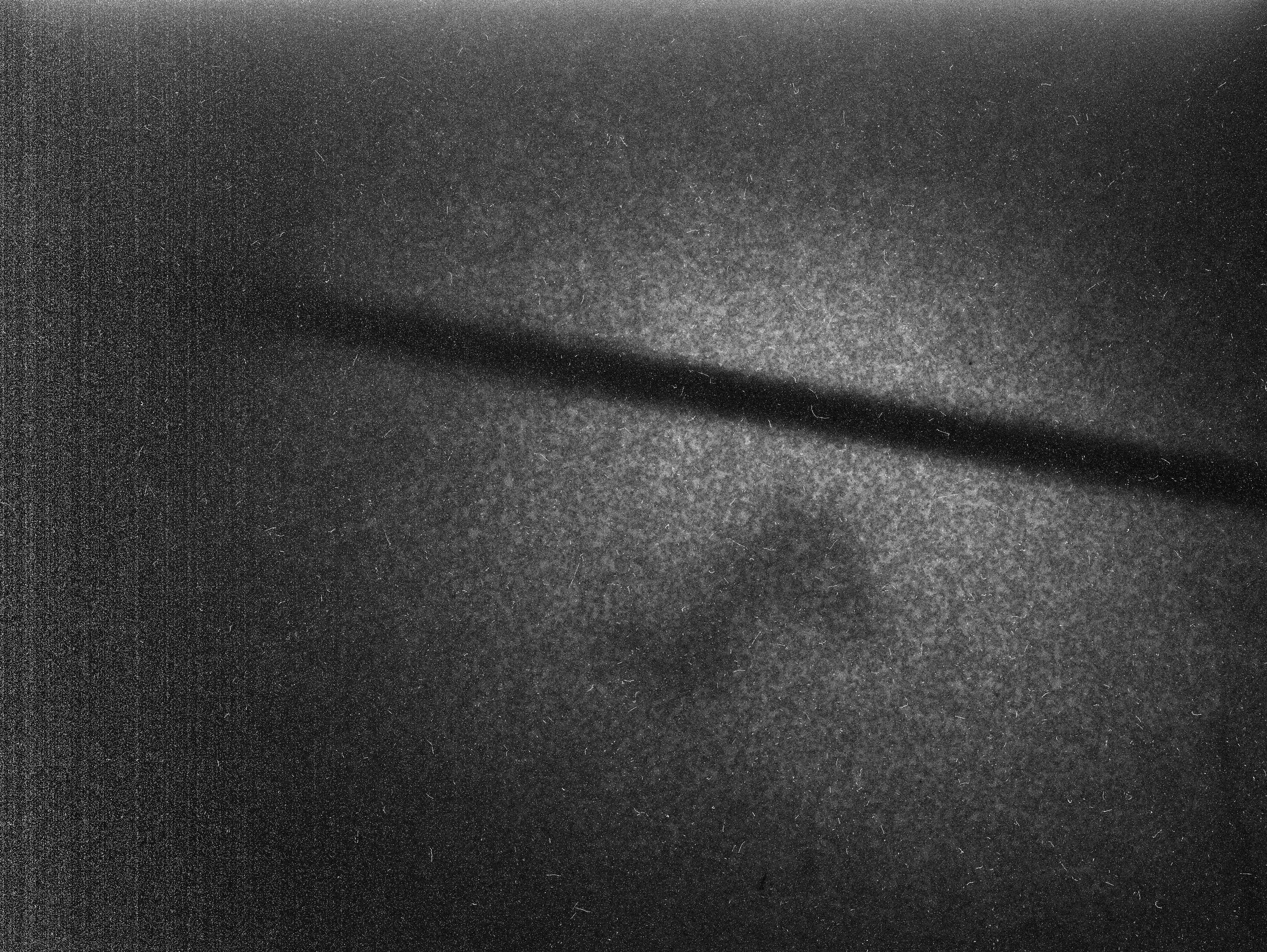

Supplement: Supplementary file 1 — Supplementary Information. [file 41598_2023_45588_MOESM1_ESM.zip › SupplementaryMaterials/PhosphorDetection3.png]

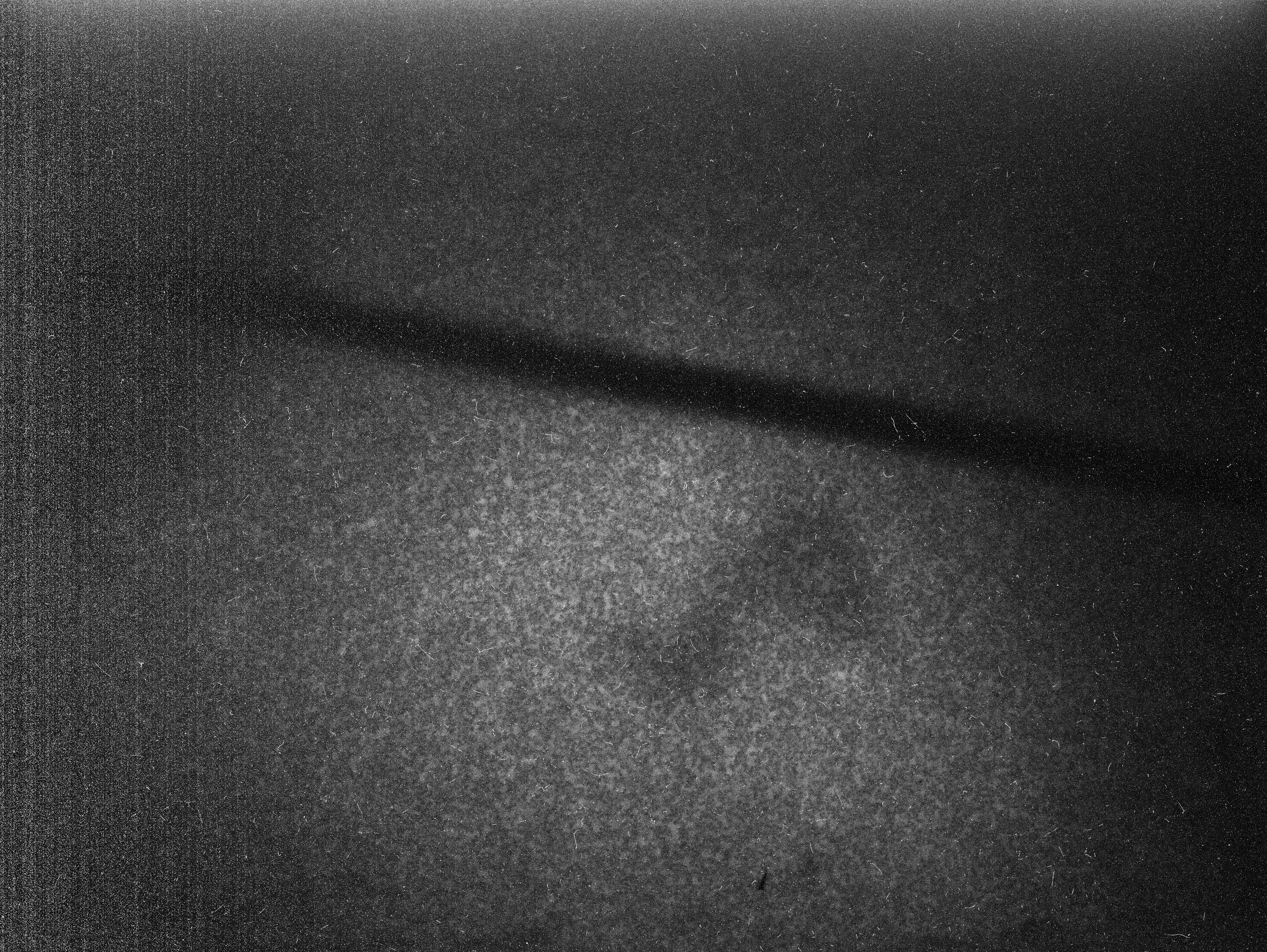

Supplement: Supplementary file 1 — Supplementary Information. [file 41598_2023_45588_MOESM1_ESM.zip › SupplementaryMaterials/PhosphorDetection4.png]

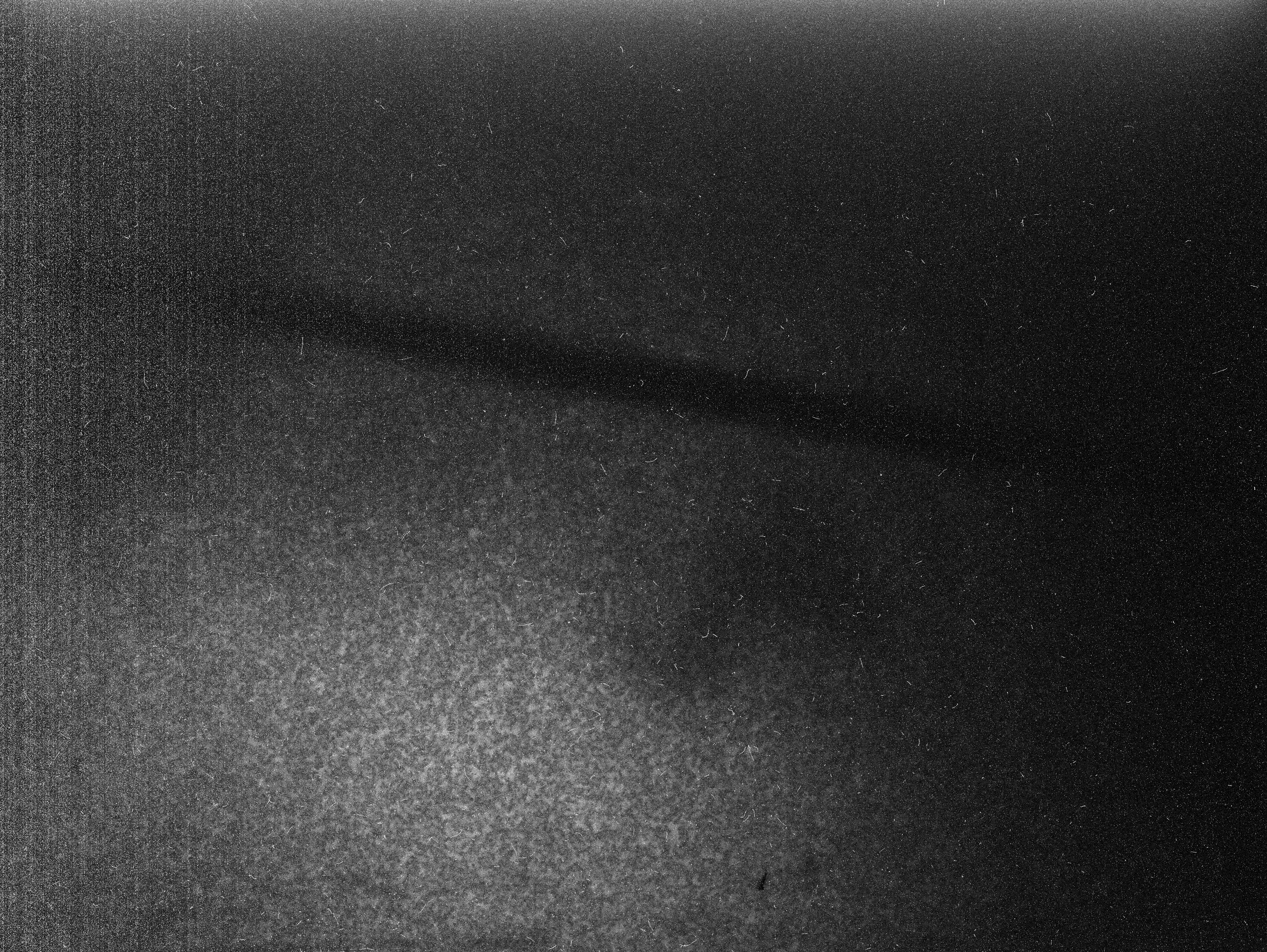

Supplement: Supplementary file 1 — Supplementary Information. [file 41598_2023_45588_MOESM1_ESM.zip › SupplementaryMaterials/PhosphorDetection5.png]

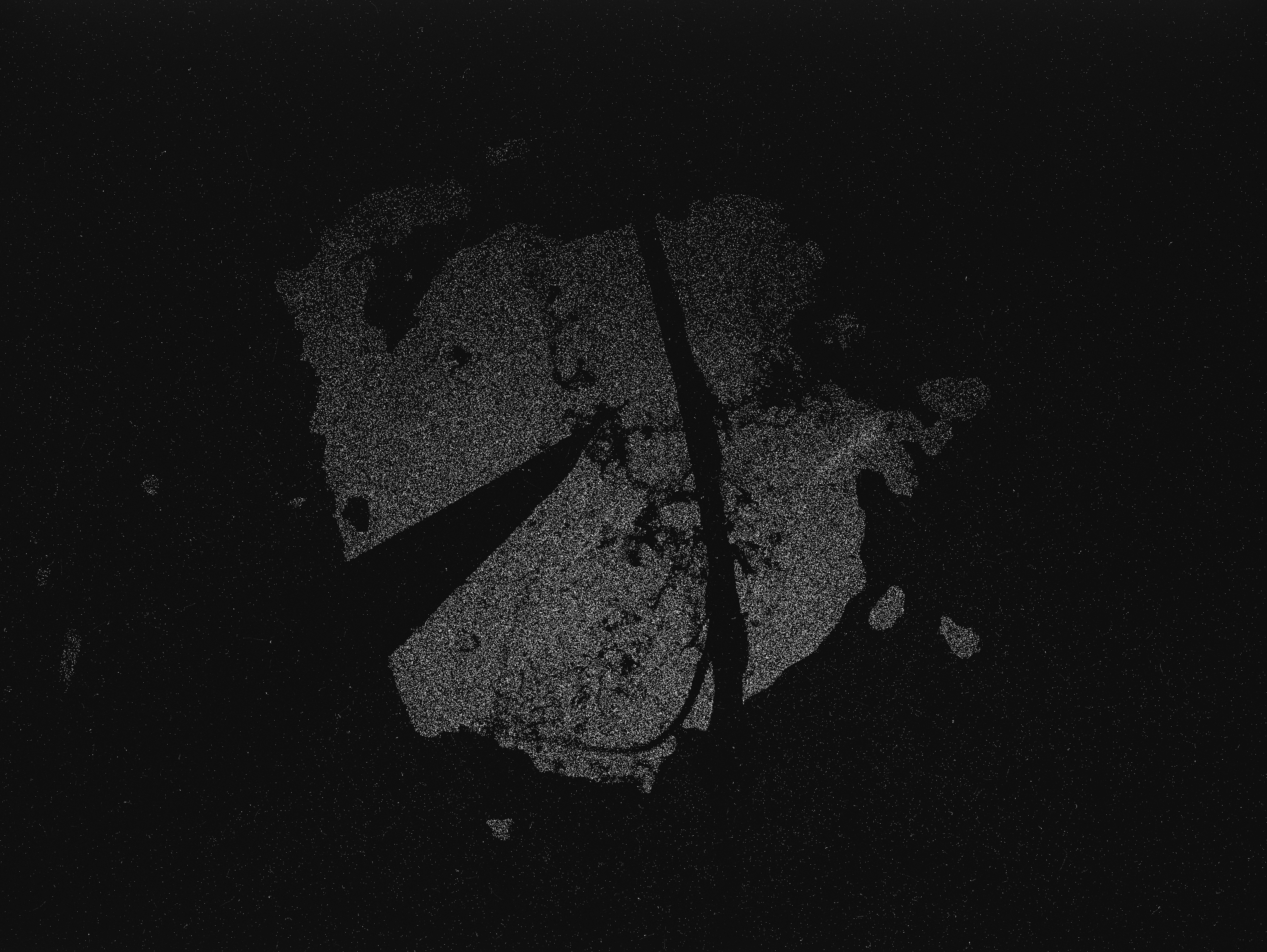

Supplement: Supplementary file 1 — Supplementary Information. [file 41598_2023_45588_MOESM1_ESM.zip › SupplementaryMaterials/DirectDetection_AlFoil_SingleFrame.png]

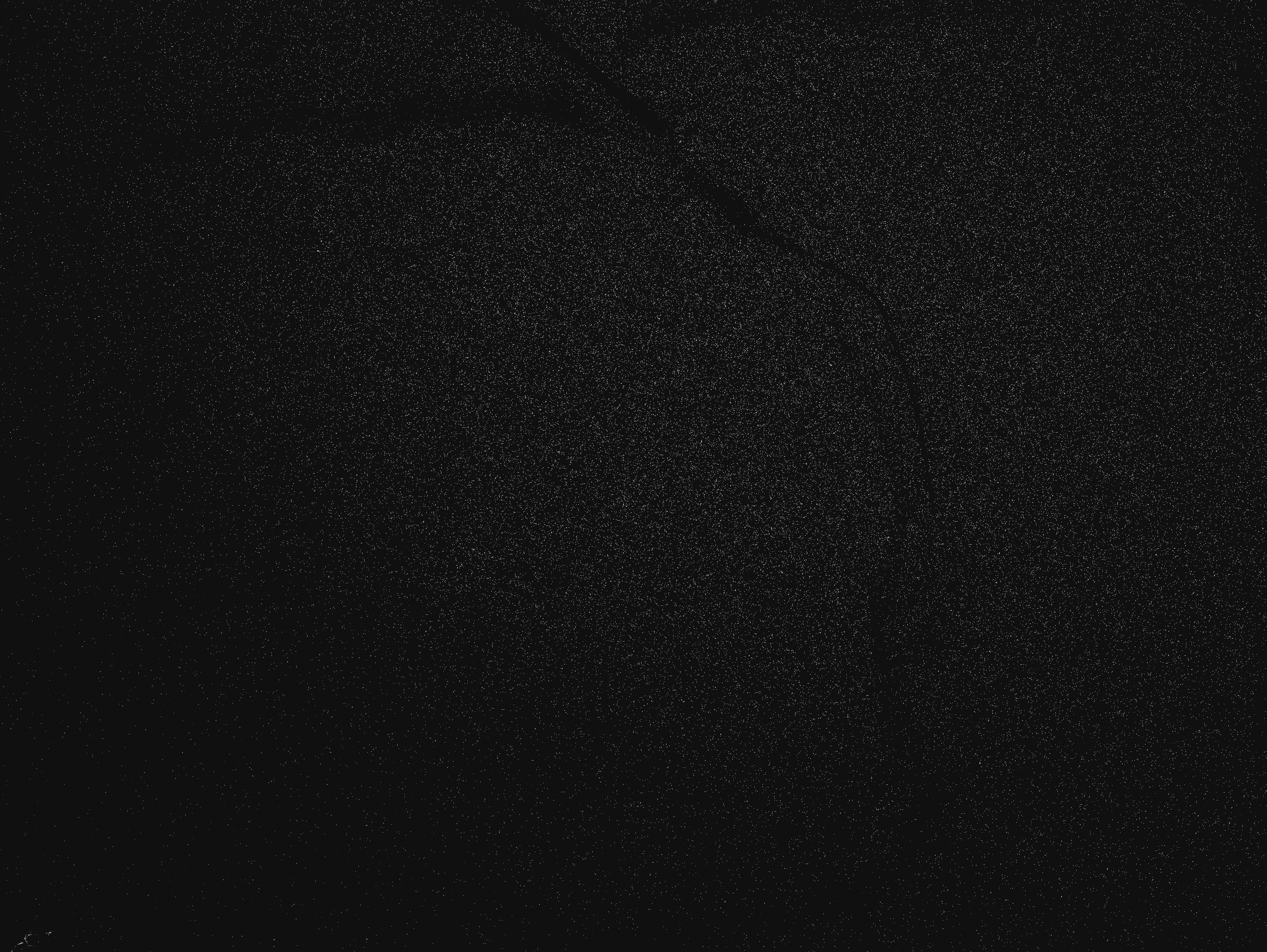

Supplement: Supplementary file 1 — Supplementary Information. [file 41598_2023_45588_MOESM1_ESM.zip › SupplementaryMaterials/DirectDetection2.png]

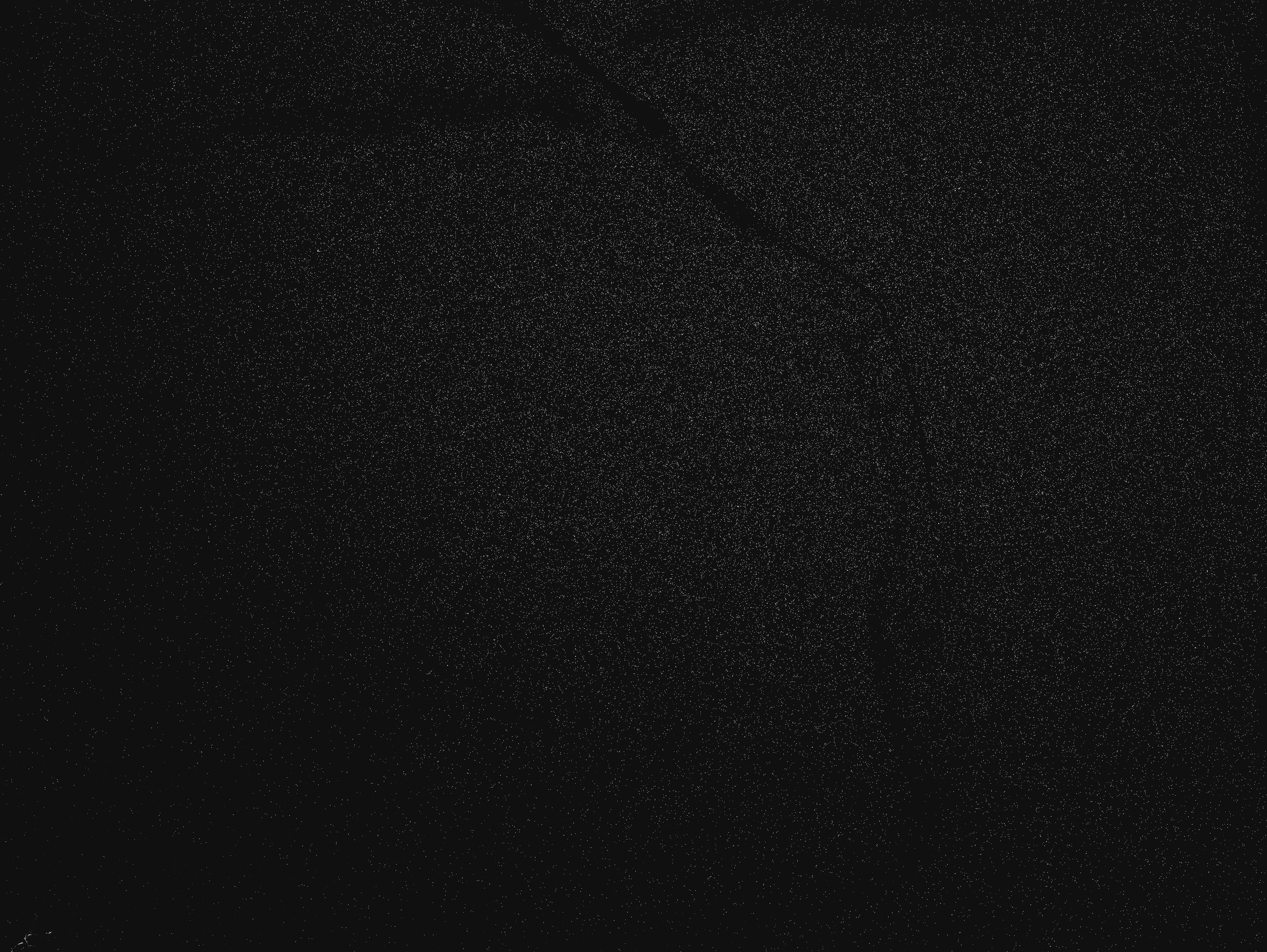

Supplement: Supplementary file 1 — Supplementary Information. [file 41598_2023_45588_MOESM1_ESM.zip › SupplementaryMaterials/DirectDetection3.png]

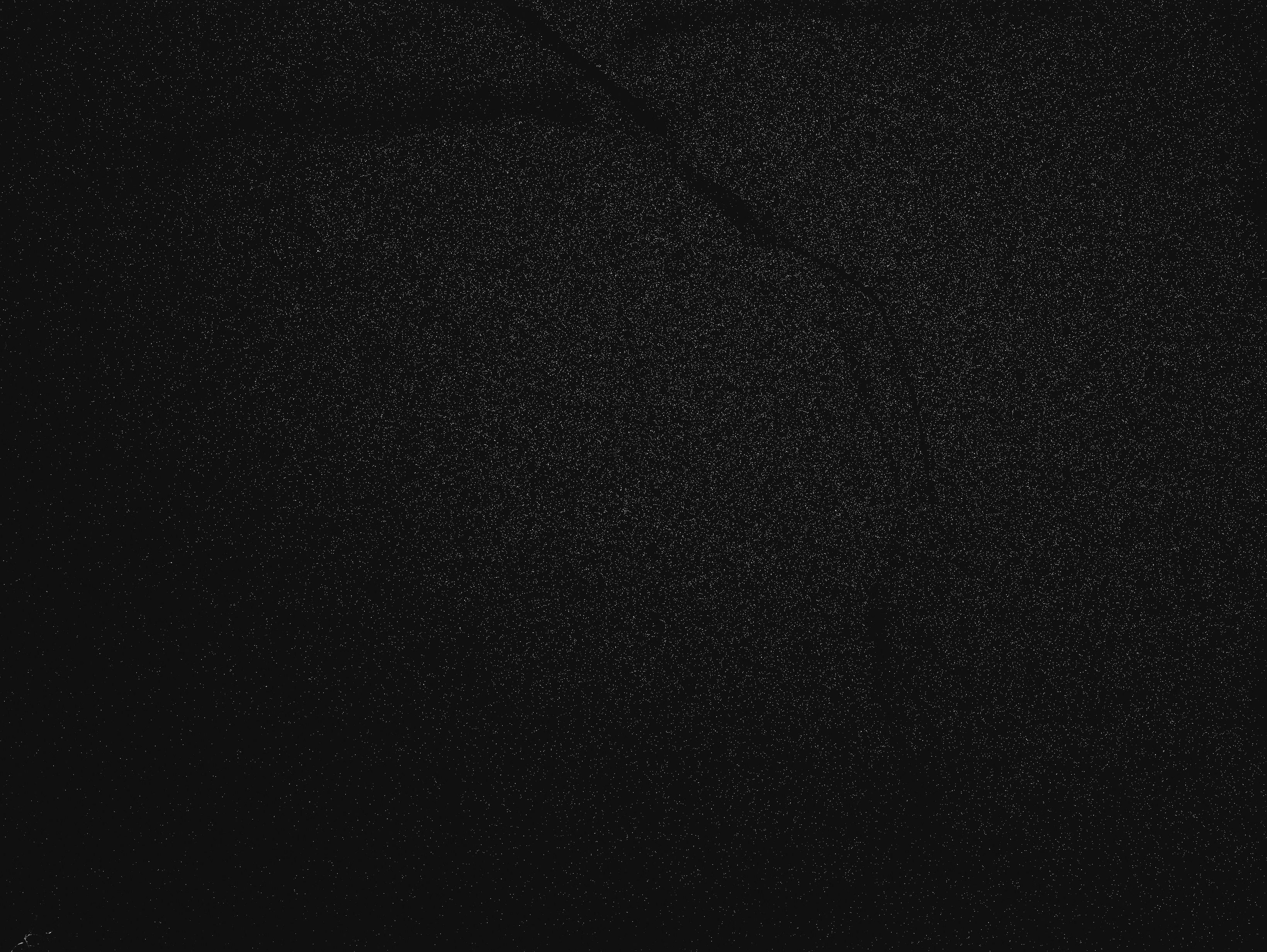

Supplement: Supplementary file 1 — Supplementary Information. [file 41598_2023_45588_MOESM1_ESM.zip › SupplementaryMaterials/DirectDetection4.png]

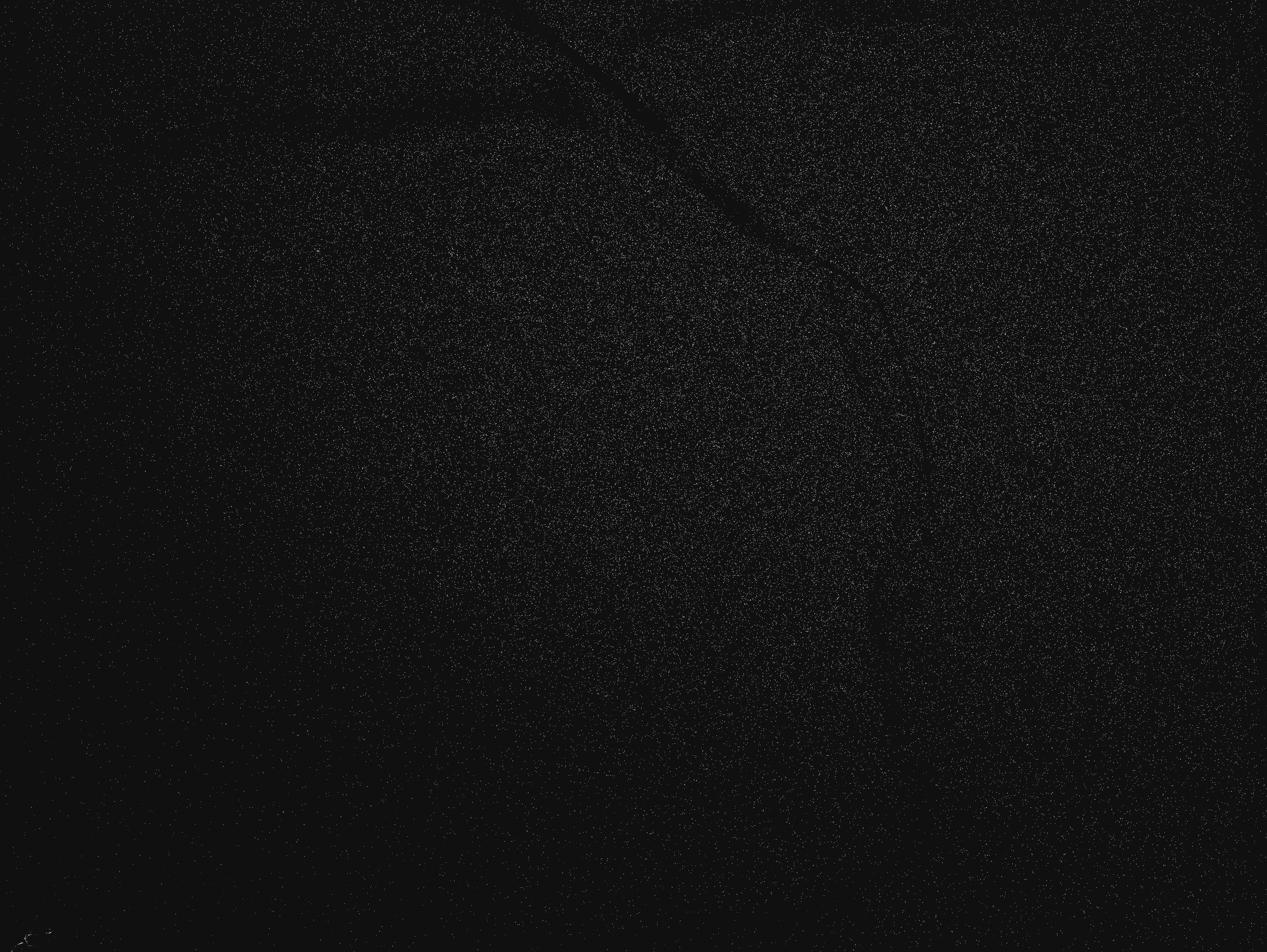

Supplement: Supplementary file 1 — Supplementary Information. [file 41598_2023_45588_MOESM1_ESM.zip › SupplementaryMaterials/DirectDetection5.png]

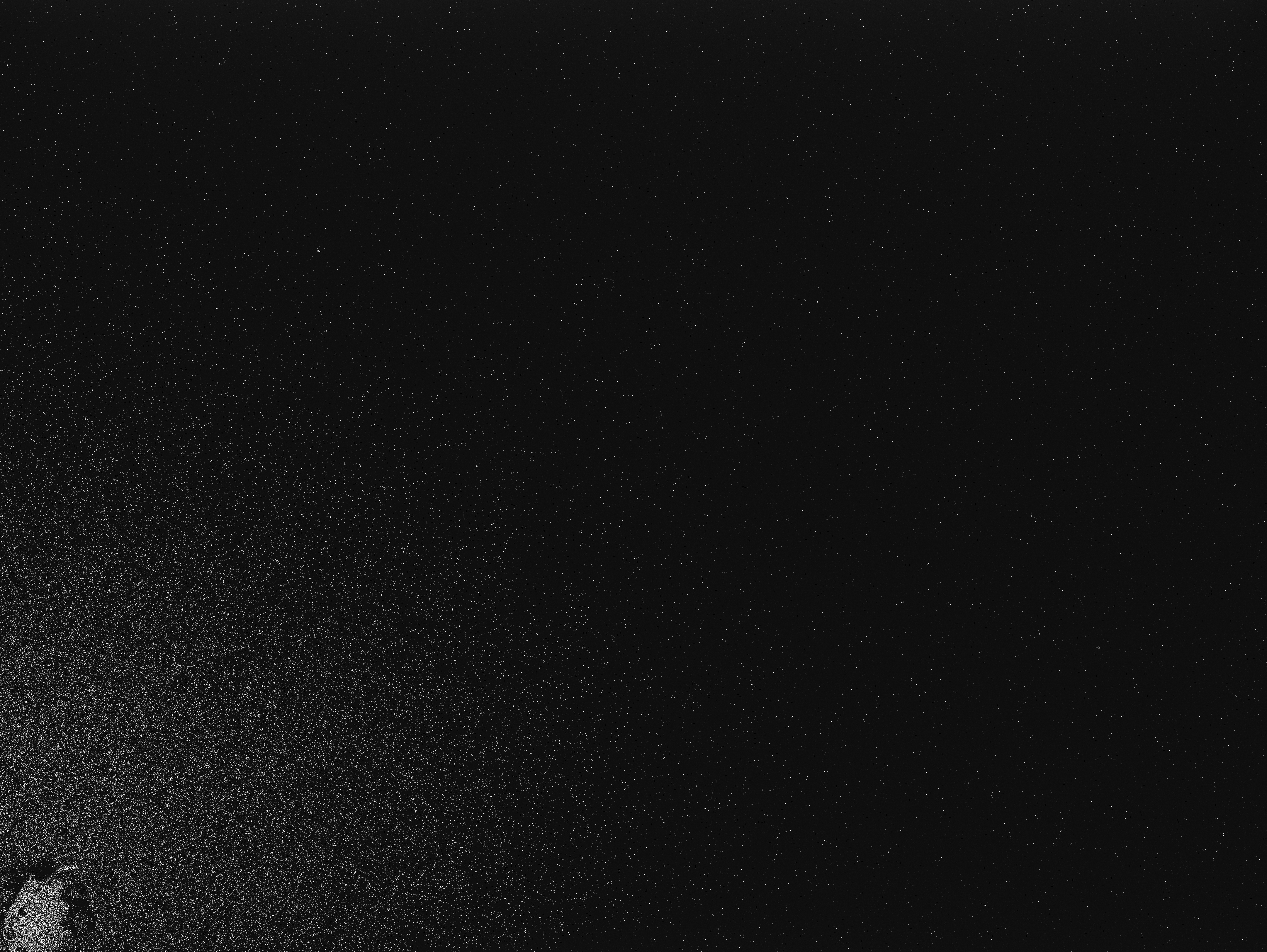

Supplement: Supplementary file 1 — Supplementary Information. [file 41598_2023_45588_MOESM1_ESM.zip › SupplementaryMaterials/AdditionalLayerRemoval.png]

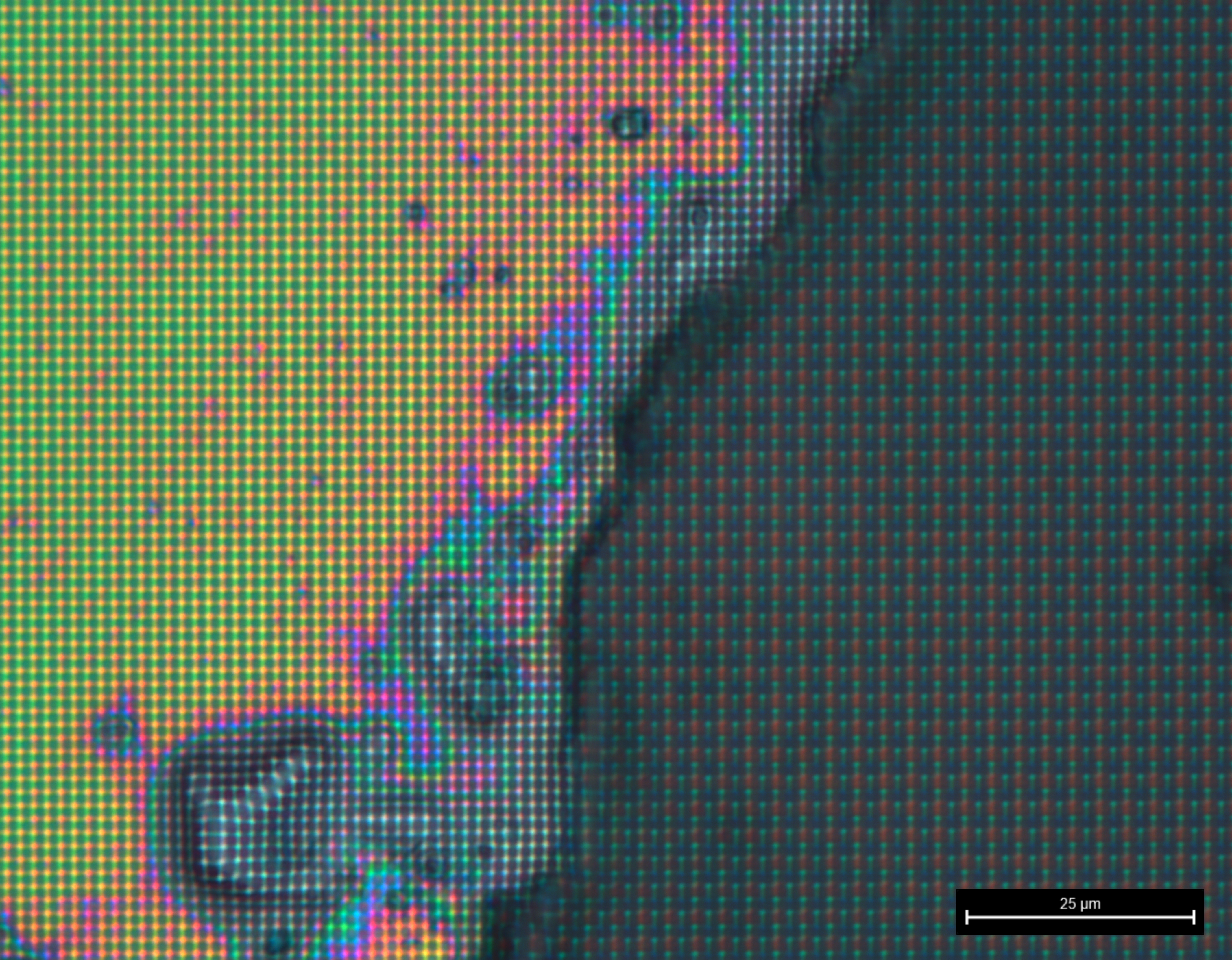

Supplement: Supplementary file 1 — Supplementary Information. [file 41598_2023_45588_MOESM1_ESM.zip › SupplementaryMaterials/PartiallyDebayeredSensor1.png]

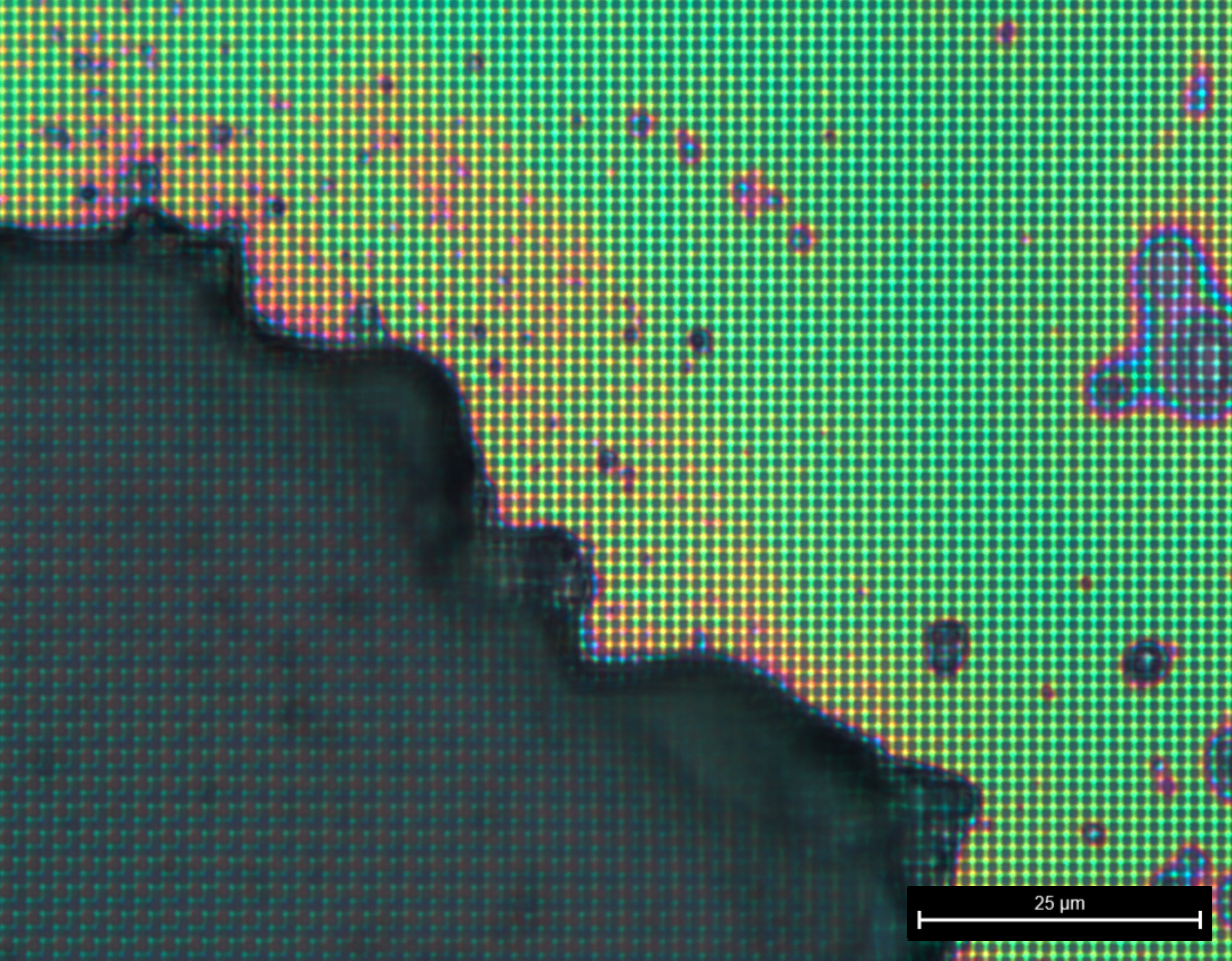

Supplement: Supplementary file 1 — Supplementary Information. [file 41598_2023_45588_MOESM1_ESM.zip › SupplementaryMaterials/PartiallyDebayeredSensor2.png]
